# Sustained intrinsic WNT and BMP4 activation impairs hESC differentiation to definitive endoderm and drives the cells towards extra-embryonic mesoderm

**DOI:** 10.1038/s41598-021-87547-7

**Published:** 2021-04-15

**Authors:** C. Markouli, E. Couvreu De Deckersberg, D. Dziedzicka, M. Regin, S. Franck, A. Keller, A. Gheldof, M. Geens, K. Sermon, C. Spits

**Affiliations:** 1 Research Group Reproduction and Genetics, Faculty of Medicine and Pharmacy, Vrije Universiteit Brussel, Brussels, Laarbeeklaan 103, Jette, 1090 Brussels, Belgium; 2 Centrum Voor Medische Genetica, UZ Brussel, Laarbeeklaan 101, Jette, 1090 Brussels, Belgium

**Keywords:** Genomic instability, Genetics, Stem cells, Embryonic stem cells, Stem-cell differentiation

## Abstract

We identified a human embryonic stem cell subline that fails to respond to the differentiation cues needed to obtain endoderm derivatives, differentiating instead into extra-embryonic mesoderm. RNA-sequencing analysis showed that the subline has hyperactivation of the WNT and BMP4 signalling. Modulation of these pathways with small molecules confirmed them as the cause of the differentiation impairment. While activation of WNT and BMP4 in control cells resulted in a loss of endoderm differentiation and induction of extra-embryonic mesoderm markers, inhibition of these pathways in the subline restored its ability to differentiate. Karyotyping and exome sequencing analysis did not identify any changes in the genome that could account for the pathway deregulation. These findings add to the increasing evidence that different responses of stem cell lines to differentiation protocols are based on genetic and epigenetic factors, inherent to the line or acquired during cell culture.

## Introduction

Human embryonic stem cells (hESCs) are a potent tool for the study of early development, in disease modelling and in regenerative medicine, as they can differentiate to any cell type of the human body. HESC differentiation protocols aim to mimic the fine orchestration of time dependent pathway modulation which is observed in vivo. Given this complexity, it is broadly acknowledged that individual cell lines may display a preference for differentiation to one germ layer over another^1,2^. This differentiation bias appears to be modulated by a wide range of factors, including genetic and epigenetic abnormalities (reviewed in^3^).

Prolonged culture affects the genetic integrity of hESC lines, resulting in point mutations^4^ and chromosomal abnormalities ranging from whole chromosome aneuploidies to smaller structural variants^3,5^. Common aneuploidies are entire or partial gains of chromosomes 1, 12, 17, and X^6–12^ while a gain of 20q11.21 is found in more than 20% of stem cell lines worldwide^6,13,14^. The selective advantage driving these abnormalities, and their potential impact on differentiation is poorly understood. As an exception, the gain of 20q11.21 leads to a *BCL2L1* dependent decrease in apoptotic sensitivity^13,14^ and an impaired TGF-β-dependent neuroectodermal commitment^15^. Furthermore, hESC carrying an extra copy of chromosome 12 proliferate faster and display a limited differentiation capacity leading to the presence of undifferentiated cells in teratomas^16^.

Epigenetic variance also plays an important role in differentiation bias^17–19^. These changes vary from DNA methylation or histone modifications^20,21^, to chromatin remodelling^22^ and X-chromosome inactivation^23,24^. For instance, mesoderm initiation requires low levels of H3K27me3 histone marks^25^ while loss of H3K4me3 facilitates neuroectoderm differentiation^26^, and decreased global non-CG DNA methylation correlates with low endodermal differentiation capacity^27^. Additionally, expression levels of miR-371-3 and other non-coding RNAs have been linked to decreased neuroectoderm differentiation efficiency^28,29^, and WNT3 levels are positively correlating with definitive endoderm commitment^30^. Lastly, *RUNX1A* affects hematopoietic lineage formation^31^ and β*FGF-1*, *RHOU* and *TYMP* are associated with low hepatic differentiation efficiency^32^.

All these findings point to differentiation bias as a complex phenomenon modulated by numerous possible genetic/epigenetic variations. In this study we investigate the mechanisms behind the loss of differentiation capacity of one of the hESC sublines in our laboratory. We identified a subline, here named VUB03_S2, with a strongly impaired differentiation capacity toward endodermal derivates, and developing an extra-embryonic mesoderm expression profile. Here we show that this is due to constitutionally hyperactivated BMP4 and WNT signalling and confirm our findings by changing cell fate in control hESC when subjecting them to the same signalling cues during definitive endoderm differentiation.

## Results

### VUB03_S2 shows impaired differentiation to neuroectoderm and differentiates to extra-embryonic mesoderm under definitive endoderm differentiation conditions

**Figure 1:**
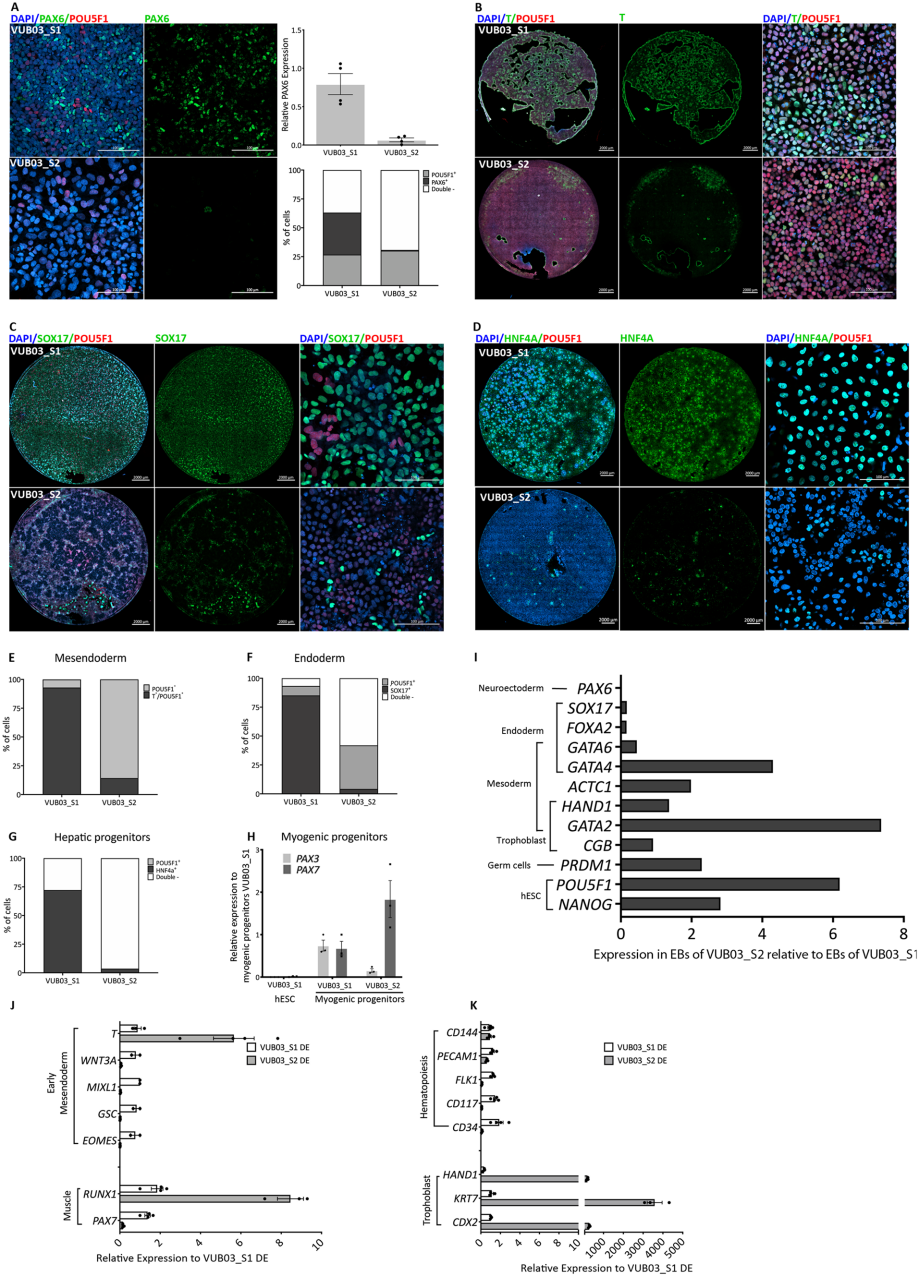
VUB03_S2 is refractory to neuroectoderm and definitive endoderm differentiation, mis-specifying to extra-embryonic mesoderm. A (left panels: immunostaining for PAX6 (green) and POU5F1 (red) in VUB03_S1 and S2 after 4 days of neuroectoderm differentiation. A (top right panel): Expression of the neuroectoderm marker *PAX6* relative to VUB03_S1 (n = 4). Data are shown as mean ± SEM, each dot represents an independent differentiation experiment and the horizontal bars with asterisks represent statistical significance between samples (P < 0.05, t-test). (**A**) (bottom right panel): counts for PAX6^+^ and POU5F1^+^ cells in neuroectoderm derived from VUB03_S1 and VUB03_S2. (**B**) Immunostaining for T (green) and POU5F1 (red) in both lines after 1-day mesendoderm induction. The left and middle panels represent full-well images. (**C**) Immunostaining for SOX17 (green) and POU5F1 (red) in both lines after 3-day definitive endoderm differentiation. The left and middle panels represent full-well images. (**D**) Immunostaining for HNF4A (green) and POU5F1 (red) in both lines after 8-day hepatoblast differentiation. (**E**–**G**) Counts of positive cells for each immunostaining. (**H**) *PAX7* and *PAX3* mRNA expression of VUB03_S2 relative to VUB03_S1, after 12-days myogenic differentiation. (**I**) mRNA expression of markers of the different germ layers in 10 days-old embryoid bodies of VUB03_S2, relative to the expression in embryoid bodies of VUB03_S1. mRNA expression of (**J**) early mesendoderm and muscle, (**K**) hematopoietic lineage and trophoblast markers in VUB03_S2 relative to VUB03_S1, after 3 days of definitive endoderm differentiation (n = 3–4). Data are shown as mean ± SEM and each dot represents an independent differentiation experiment. Scale bars represent 2000 μm and 100 μm.

VUB03_S2 is a genetically abnormal hESC sub-line in our lab, that has acquired a gain of 20q11.21. The line shows the characteristic gene-expression profile of a pluripotent stem cell line, including expression of *NANOG*, *POU5F1*, *TRA-160* and *TRA-1-81*. While expression of NANOG at the mRNA level was significantly higher in VUB03_S2 than in VUB03_S1, no differences were visible at the protein level by immunostaining (Supplementary Fig. S1). We first assessed the differentiation capacity of VUB03_S2 to neuroectoderm, as compared to its genetically normal counterpart VUB03_S1. As expected, VUB03_S2 differentiated poorly to neuroectoderm after a 4-day induction protocol using a dual TGF-β inhibition^33,34^. We observed a 140-fold higher *PAX6* mRNA expression in VUB03_S1-derived cells and 0.65% PAX6^+^ cells in the case of VUB03_S2 (Fig. 1A). These results are in line with our work showing that hESC with a gain of 20q11.21 have a TGF-β-dependent impaired neuroectoderm commitment^15^.

Next, we assessed the differentiation to mesendoderm through a 24 h induction that mimics the first steps of primitive streak formation, by WNT and TGF-β activation. We found that 93% of VUB03_S1 cells versus 14% of VUB03_S2 are positive for T, an early mesoderm marker, while for both lines most cells are still also POU5F1^+^ (Fig. 1B,E). These results show that while VUB03_S1 readily responds to the cues for mesendoderm induction, VUB03_S2 shows a delayed induction of T.

We then performed a 3-day differentiation to definitive endoderm through WNT and TGF-β activation for the first 24 h and only TGF-β activation thereafter. VUB03_S1 efficiently differentiates to endoderm, with 85% of cells being SOX17^+^, while VUB03_S2 shows 4% of correctly differentiated cells. Eight percent of VUB03_S1 cells and 38% of VUB02_S2 were POU5F1^+^ (Fig. 1C,F). To assess if this impairment remained in long-term differentiation, we further differentiated the cells to hepatic progenitors in an 8-day differentiation protocol. The immunofluorescence results for HNF4A (Fig. 1D) and cell counts (Fig. 1G) support that here too VUB03_S2 fails to differentiate to the endodermal lineage, while exiting the pluripotent state.

To assess whether this deficiency extended to the mesoderm lineage, we performed a 12-day induction to myogenic progenitors with myogenic medium supplemented with CHIR99021 (WNT signalling activator) for the first 48 h and subsequently by FGF2 for the remainder of the differentiation. VUB03_S1 and VUB03_S2 were able to differentiate similarly to myogenic progenitors (Fig. 1H), with over 5000-fold induction of *PAX3* and 200-fold increase in *PAX7* expression in both lines, as compared to undifferentiated cells.

Finally, we spontaneously differentiated both lines into embryoid bodies and quantified the expression of transcripts associated to neuroectoderm, endoderm, mesoderm, trophoblast, germ cells and the pluripotent state (Fig. 1I). In line with the results of the directed differentiation, VUB03_S2 showed reduced neuroectoderm induction (associated to its gain of 20q11.21), and lower expression of endoderm markers *SOX17* and *FOXA2* as compared to VUB03_S1. Expression of genes associated to the other lineages were equally or higher expressed than in VUB03_S1, and the embryoid bodies obtained from VUB03_S2 showed higher levels of *POU5F1* and *NANOG* than those obtained from VUB03_S1.

Taken together, the results show that VUB03_S2 is refractory to neuroectoderm and definitive endoderm differentiation. Also, VUB03_S2 did not remain undifferentiated, as indicated by the low expression of *POU5F1* in the differentiation protocols beyond day 1. In order to investigate the alternative cell fate reached by VUB03_S2 upon definitive endoderm differentiation, we assessed a variety of markers for different fates after 3 days of definitive endoderm differentiation: early mesendoderm (*T, WNT3A, MIXL1, GSC, EOMES*), muscle (*RUNX1, PAX7*), hematopoietic lineage (*CD144, PECAM, FLK1, CD117, CD34*) and trophoblast (*HAND1, KRT7, CDX2*)(Fig. 1J,K), as well as in the undifferentiated cells (Supplementary Fig. S2). VUB03_S2 showed a five- to eightfold higher expression of mesoderm markers *T* and *RUNX1* and a 500–3500 times higher expression of the trophoblast markers. Only *HAND1* showed a higher basal level in the undifferentiated VUB03_S2 as compared to VUB03_S1 (Supplementary Fig. S2). The protein levels of KRT7 were also found to be elevated (Supplementary Fig. S3). Taken together, these results show that VUB03_S2 does not respond as expected to the definitive endoderm differentiation cues, and the combination of mesoderm and trophoblast marker expression suggests that it differentiates to extra-embryonic mesoderm instead.

### VUB03_S2 shows a distinct transcriptomic profile with a deregulation of BMP4 and WNT signalling

**Figure 2:**
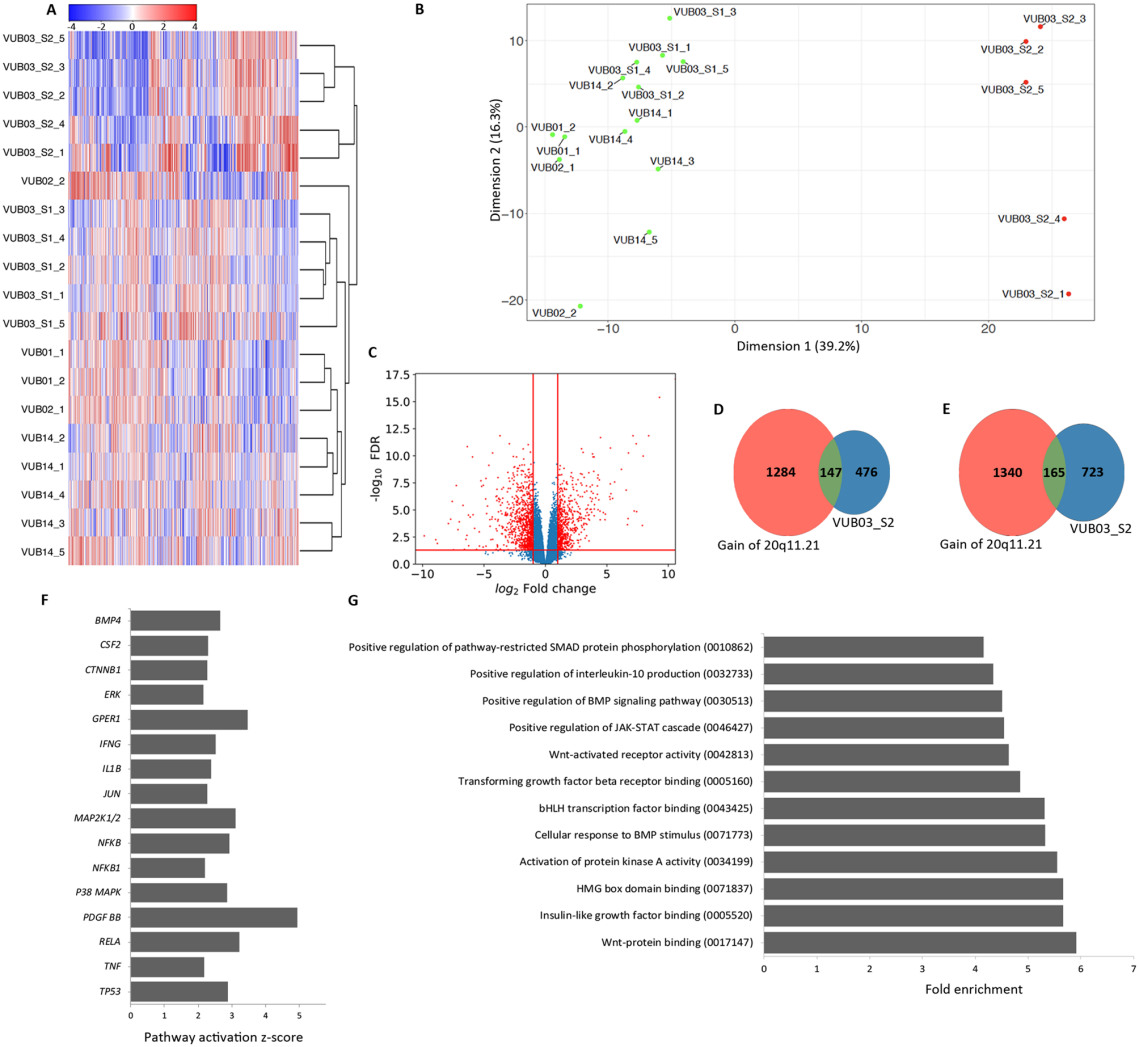
VUB03_S2 shows a unique transcriptomic signature characterized by WNT and BMP4 signalling deregulation. (**A**) Unsupervised cluster analysis and (**B**) Principal component analysis of component 1 versus component 2 of all coding genes with a count per million greater than one in at least two samples. (**C**) Volcano plot of the differential gene expression analysis of VU03_S2 versus all other hESC lines. The red lines show cut-off values of |log_2_ Fold Change|> 1 and FDR < 0.05. (**D**,**E**) Venn diagrams comparing the deregulated genes in VUB03_S2 with those deregulated in lines with a 20q11.21 gain. (**D**) Shows the upregulated genes and (**E**) the downregulated genes (**A**–**E**)^35^. (**F**) Ingenuity pathway analysis of all differentially expressed genes with |log_2_ Fold Change|> 1 and FDR < 0.05. The plot shows only upstream regulators of pathways with an activation score below -2 and above 2 with a p < 0.05. (**G**) DAVID Enrichment Analysis of the 1000 top-deregulated genes, showing only gene-ontology terms related to intracellular signalling (full list in supplementary table S2). In parenthesis are the gene-ontology term numbers.

In order to investigate the mechanisms behind the lack of ability to differentiate to definitive endoderm derivates, we carried out a transcriptome analysis by RNA sequencing on VUB03_S2 and four control lines with proven ability to differentiate to all three germ layers (VUB01, VUB02, VUB03_S1 and VUB14, differentiation data for VUB01 and VUB02 published in Markouli et al., 2019; data for VUB14 not shown). We included two to five replicates per line, obtained from samples collected from independent cell cultures. Only coding genes with a count per million greater than one in at least two samples were included in our analysis. In unsupervised hierarchical clustering, all five replicates of VUB03_S2 cluster together and apart from the other four lines, including VUB03_S1 (Fig. 2A). The principal component analysis plot of all expressed genes shows the same pattern, with the five VUB03_S2 replicates organizing in a common cluster that is distinct from the control lines (Fig. 2B). This illustrates that the transcriptome of VUB03_S2 differs significantly from the other tested lines. Differential gene expression analysis with a cut-off value of |log_2_ Fold Change|> 1 and false discovery rate q-value (FDR) < 0.05 shows 623 up-regulated and 888 down-regulated genes in VU03_S2 versus control lines (Fig. 2C). A list with the top-50 differentially expressed genes can be found in the supplementary Table S1.

To ensure that these differences were not the result of the gain of 20q11.21 present in VUB03_S2, we compared the up- and down-regulated genes found in VUB03_S2 to those identified in other lines with a gain of 20q11.21 (Fig. 2D,E respectively, data published in Markouli et al. 2019). Thirty percent of the upregulated genes and 22% of the downregulated genes in VUB03_S2 are in common to those deregulated in lines with a gain of 20q11.21. This limited transcriptomic similarity between the two groups shows that VUB03_S2 displays a unique transcriptome that is only partially related to the chromosomal abnormality it carries.

We used Ingenuity Pathway Analysis^36^, using all genes with a |log_2_ Fold Change|> 1 and FDR < 0.05, to predict the activation state of upstream pathway regulators. We considered only pathways with p-value < 0.05 and |z-score|> 2, which are predicted by 10 or more regulators to be significantly activated or inhibited. (Fig. 2F). We also carried out functional annotation enrichment analysis of the top-1000 deregulated genes using DAVID. This retrieved a list of 558 annotations, of which we selected the gene ontology terms with a fold-enrichment > 4, and a p-value < 0.05. The list of gene ontology terms referring to intracellular signalling is shown in Fig. 2G, the full list of 49 terms can be found in the supplementary Table S2. Based on the common predictions retrieved from both tools, we could conclude that VUB03_S2 shows differentially regulated pathways that are key to the control of differentiation such as BMP4 and WNT (beta-catenin). Finally, we inspected the expression of 15 WNT targets (http://web.stanford.edu/group/nusselab/cgi-bin/wnt/target_genes) and 64 SMAD1 target genes (downstream effector of BMP4, http://chip-atlas.org/target_genes) in VUB03_S2 as compared to VUB03_S1 and to three other genetically balanced hESC lines (VUB01, VUB02 and VUB14). We found that 9 out of the 15 WNT targets were upregulated in VUB03_S2 as compared to the other lines (Supplementary Fig. S4) and 24 out of 64 SMAD1 target genes were deregulated in VUB03_S2 (Supplementary Fig. S5).

### The transcriptomic profile of VUB03-S2 cannot be explained by additional chromosomal abnormalities or exomic point mutations

As mentioned above, VUB03_S2 carries a 4 Mb gain of 20q11.21, with no further de novo gains or losses in other chromosomes. Supplementary Table S3 contains the karyotypes of all lines used in the study. The gain of 20q11.21 in VUB03_S2 unlikely explains the phenotype since in our lab we have studied multiple hESC lines with smaller and larger gains of 20q11.21 and with higher copy numbers, none of which showed signs of poor definitive endoderm commitment (Markouli et al., 2019). This suggests that VUB03_S2, although karyotypically similar to these lines, presents a unique differentiation bias unrelated to its karyotype.

In order to investigate if de novo point mutations could be responsible for the transcriptomic changes observed in VUB03_S2, we carried out whole exome sequencing on bulk DNA samples of VUB03_S1 and VUB03_S2. We first filtered the variants for de novo appearance in VUB03_S2 and by only considering those with a read depth > 10. Recurrent sequencing artefacts were avoided by omitting changes that are found > 100 times in the exome sequencing database of our sequencing facility. Finally, we only considered variants that were present in at least 30% of reads, to only include variants that were fixed in the population in at least a heterozygous state. This yielded 917 variants, of which 849 were in non-coding regions and 68 in protein coding sequences (Supplementary Table S4 shows an overview of the location of the variants). Fifty-two of the protein-coding variants were non-synonymous. The exact location and nature of these variants are listed in the Supplementary Table S5. We annotated the functions of the genes using the public databases NCBI-Gene and GeneCards, and found that none of the variants was located in a gene with a link to the WNT and BMP4 signalling.

Alternatively, the loss of a transcription factor binding site could potentially explain changes in gene-expression and pathway activation. As such we evaluated the possibility that a variant had negatively impacted transcription factor binding sites in *BMP4*, *CTNNB1* and their downstream effectors and targets (list obtained from the Ingenuity Pathway Analysis database, 198 and 535 genes for *BMP4* and *CTNNB1,* respectively). We found that 13 of these genes contained a de novo variant (Supplementary Table S6). We used cistrome^37^ to identify the transcription factors binding the regions containing the variants and PWMScan to establish if the variant abolished the transcription binding site^38^. None of the variants resulted in the destruction of transcription factor binding sites. Because of the use of exome sequencing data, we could only assess a limited number of transcription binding sites. Therefore, it cannot be excluded that the subline carries variants in regulatory regions not covered by our sequencing approach.

### Sustained intrinsic WNT and BMP4 activation impairs differentiation to definitive endoderm and drives the cells towards extra-embryonic mesoderm

**Figure 3:**
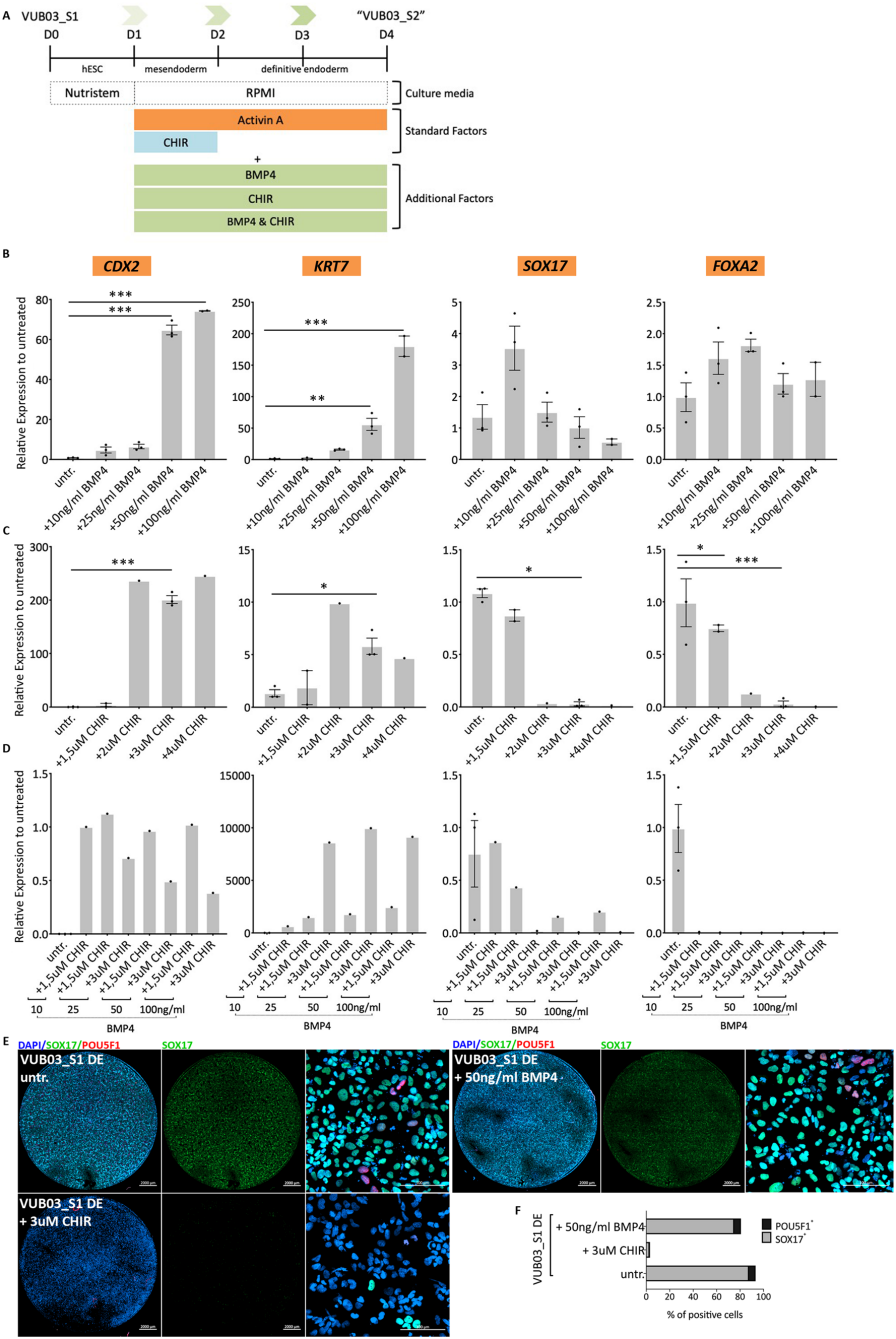
Impairing the definitive endoderm differentiation of VUB03_S1 through WNT and BMP4 pathway activation. (**A**) A schematic representation of the experimental design. WNT and BMP4 activators, in different concentrations, are added to the normal definitive endoderm differentiation medium. (**B**–**D**) mRNA expression of trophoblast markers *CDX2, KRT7* and definitive endoderm markers *SOX17* and *FOXA2* in VUB03_S1 after definitive endoderm differentiation in different treatment conditions. The data is plotted relative to VUB03_S1 differentiated in standard conditions (untreated cells)*.* (**B**) After use of BMP4 only. (**C**) After use of CHIR99021 only. (**D**) Combination of both factors (n = 1–3). Data are shown as mean ± SEM, each dot represents an independent differentiation experiment. (**E**) Immunostaining for SOX17 (green) and POU5F1 (red) and (**F**) counts for SOX17 and POU5F1-positive cells in VUB03_S1 after 3 days of definitive endoderm differentiation untreated and after BMP4 and CHIR treatment. Scale bars represent 2000 μm and 100 μm. For those conditions where multiple replicates were available, statistical significance was tested by 1-way Anova and Bonferroni’s multiple comparison test. *Indicates p < 0.01, **p < 0.001, ***p < 0.0001.

The transcriptomic analysis predicted dysregulation of the WNT and BMP signalling, both are key regulators of differentiation and lineage commitment. A brief WNT activation is necessary to induce definitive endoderm differentiation, and BMP4 is key in extra-embryonic mesoderm specification^39^. Therefore, we aimed at validating the implication of these pathways in the misspecification observed in VUB03_S2. First, we mimicked the phenotype by subjecting control lines to the standard differentiation media, supplemented with recombinant BMP4 and the WNT activator CHIR99021 (experimental setup illustrated in Fig. 3A). The identification of the optimal concentrations of factors was established using VUB03_S1, and their effect validated on two additional control lines.

First, we exposed the cells to exogenous BMP4 during definitive endoderm differentiation, in four different concentrations: 10 ng/ml, 25 ng/ml, 50 ng/ml and 100 ng/ml (Fig. 3B). We evaluated mRNA levels for *CDX2* and *KRT7* to determine if the cells were becoming trophoblast-like and *SOX17, FOXA2* to evaluate definitive endoderm. While the addition of BMP4 did not affect the expression levels of the specific definitive endoderm markers, *CDX2* and *KRT7* were induced at high concentrations (63 and 75-fold increase as compared to the untreated cells). These findings suggest that BMP4 activation is necessary for the cells to acquire an extra-embryonic mesoderm expression profile while having little effect on the expression of DE markers. This is further illustrated in Fig. 3E,F where the SOX17 protein levels between the untreated and BMP4-treated VUB03_S1 show minimal differences.

We then tested the effect of WNT activation using different concentrations of CHIR99021 (1.5, 2, 3 and 4 μM, Fig. 3C). While the lowest concentration (1.5 μM) of CHIR did not show a noticeable effect compared to the untreated line, the three higher concentrations (2, 3 and 4 μM) resulted in a significant induction of *CDX2* expression (200-fold increase), a modest increase in the levels of *KRT7* (sevenfold increase) and strong decrease in *SOX17* and *FOXA2* (80–300-fold decrease). Nevertheless, although *KRT7* was up-regulated in comparison to the untreated cells, the induction was lower than what we observed after the addition of BMP4. The effect on definitive endoderm induction was also demonstrated at the protein level in Fig. 3E,F, with a strong decrease of SOX17^+^ cells in the CHIR-treated condition compared to the untreated.

When we combined both factors (Fig. 3D), we found that the combination of 3 μM of CHIR and any amount of BMP4 between 25 and 100 ng/mL resulted in a loss of endoderm specification and a differentiation into extra-embryonic mesoderm. This suggests that WNT activation has the strongest effect on the impairment of differentiation to definitive endoderm while BMP4 is mostly responsible for the *KRT7* up-regulation, revealing a synergistic effect of the two pathways to explain the phenotype of VUB03_S2. We confirmed these results on two additional karyotypically normal lines (VUB02 and VUB19), treating them with 3 μM CHIR and 50 ng/mL BMP4 (Supplementary Figs. S6 and S7).

**Figure 4:**
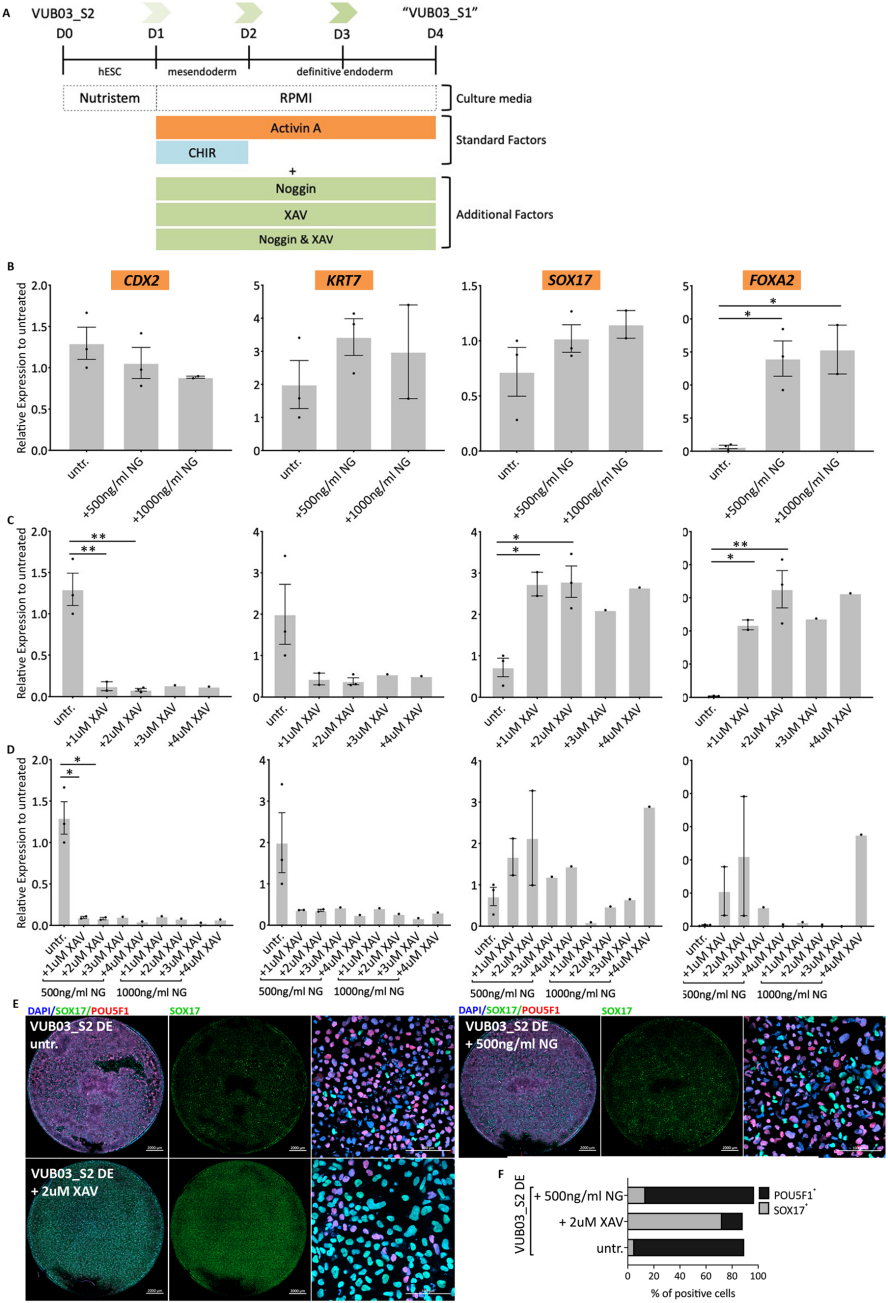
Restoration of the definitive endoderm differentiation capacity of VUB03_S2 through WNT and BMP4 pathway inhibition. (**A**) A schematic representation of the experimental design. WNT and BMP4 inhibitors, in different concentrations, are added to the normal definitive endoderm differentiation (**B**–**D**) mRNA expression after definitive endoderm differentiation of trophoblast markers *CDX2, KRT7* and definitive endoderm markers *SOX17* and *FOXA2* in VUB03_S2 after definitive endoderm differentiation in different treatment conditions. The data is plotted relative to VUB03_S2 differentiated in standard conditions (untreated cells)*.* (**B**) After use of Noggin only. (**C**) After use of XAV-939 only. (**D**) Combination of both factors (n = 1–3). Data are shown as mean ± SEM, each dot represents an independent differentiation experiment. (**E**) Immunostaining for SOX17 (green) and POU5F1 (red) and (**F**) counts for SOX17 and POU5F1-positive cells in VUB03_S2 untreated / Noggin and XAV- treated cells after 3 days of definitive endoderm differentiation. Scale bars represent 2000 μm and 100 μm. For those conditions where multiple replicates were available, statistical significance was tested by 1-way Anova and Bonferroni’s multiple comparison test. *Indicates p < 0.01, **p < 0.001, ***p < 0.0001.

Finally, to validate our findings, we aimed to rescue the definitive endoderm differentiation capacity of VUB03_S2 by exposure to noggin (NG, BMP4 pathway inhibitor) and XAV-939 (WNT pathway inhibitor). The experimental design is shown in Fig. 4A. At the concentrations tested, Noggin did not inhibit the induction of *CDX2* and *KRT7* but did increase the expression of *SOX17* (9 to 19-fold) while the effect on *FOXA2* was modest (1.5- to 2-fold increase, Fig. 4B). On the other hand, treatment with XAV-939 resulted in 3- to 19-fold decrease in extra-embryonic mesoderm markers and a significant improvement in the definitive endoderm differentiation (Fig. 4C) while combination of both factors showed a similar outcome (Fig. 4D). The effect of each compound on DE induction can be also seen in the immunostaining images for SOX17, in VUB03_S2, Noggin and XAV-treated cells (Fig. 4E). Cell counts for SOX17^+^ and POU5F1^+^ cells (Fig. 4F) suggest that WNT inhibition of VUB03_S2 with XAV-939 rescues its DE deficiency and restores SOX17 levels comparable to VUB03_S1 untreated cells (Fig. 3F).

## Discussion

The aim of our study was to elucidate the cause of the impaired differentiation capacity of VUB03_S2. We previously showed that the gain of 20q11.21 this subline carries causes the failure to differentiate to neuroectoderm^15^, but this could not explain its reduced definitive endoderm differentiation capacity. Moreover, this subline is able to exit pluripotency, and to preferably yield extra-embryonic mesoderm cells. In this study we demonstrate that intrinsically hyperactivated WNT and BMP4 signalling are responsible for this atypical response to definitive endoderm differentiation cues. Moreover, we were able to reverse this response by the use of WNT and BMP4 inhibitors.

To the best of our knowledge, this is the first report on a human pluripotent stem cell (hPSC) line that is not only showing differentiation impairment, but also specification to the wrong lineage when submitted to well-established differentiation cues. Several groups have reported that the expression levels of specific genes may modulate the propensity of a line to a specific lineage, but in none of these cases the lines committed to a completely different lineage^28–32^. Here, we find that WNT and BMP4 activation, along with TGF-β activation by Activin A results in extra-embryonic mesoderm formation. This finding is not entirely unexpected, as it is known that *CDX2* is a downstream target of WNT signaling^40,41^. Furthermore, BMP4 signaling is required for the induction extraembryonic mesoderm^39^.

While we could demonstrate the constitutional activation of these pathways resulting in the abnormal response to the differentiation cues, we could not identify its genomic cause. VUB03_S2 did not carry additional de novo chromosomal abnormalities or point mutations that could explain its transcriptomic profile, suggesting that the cause may either be found in the genomic regions that are not covered by the exome sequencing or potentially in epigenetic modifications. A number of studies have established a link between epigenomic changes and differentiation bias^25,42^, although the mechanistic link remains to be unraveled.

Many current differentiation protocols suffer from reproducibility issues between laboratories and lines. While this is commonly thought to be a result of suboptimal protocols, it is becoming increasingly clear that individual lines are more likely at the origin. Our work here shows in detail how hESC can acquire pervasive deregulation of intrinsic differentiation signals, thereby losing their ability to respond adequately to well established differentiation triggers. Furthermore, while a growing body of work has begun to demonstrate that failure to differentiate to certain lineages can be explained by commonly recurring mutations, often such outcomes may have no clear driver, as seen here, which may lead to the false conclusions on differentiation protocol reproducibility and general experimental variability. As hPSC are increasingly moving towards clinical trials, line choice will become increasingly important, and a comprehensive evaluation of differentiation biases will be vital to their long-term success.

## Experimental procedures

### hESC lines and culture

HESC were derived and characterized as previously described^43,44^. All lines are registered with the EU hPSC registry (https://hpscreg.eu/). hESC were cultured on dishes coated with 10 μg/ml laminin-521 (Biolamina) in NutriStem hESC XF medium (NS medium; Biological Industries) with 100 U/ml penicillin/streptomycin (Thermo Fisher Scientific) and passaged as single cells in a 1:10 to 1:100 ration using TrypLE Express (Thermo Fisher Scientific) when 70–80% confluent.

### Differentiation to neuroectoderm

The protocol was adapted from^33^ and^34^. Differentiation was carried out on laminin-521 and initiated when the hESC culture was 90% confluent. The neurectoderm differentiation medium consisted of KnockOut D-MEM (Thermo Fisher Scientific), 10% KnockOut Serum Replacement (Thermo Fisher Scientific), 500 ng/ml Recombinant Human Noggin Protein (R&D Systems) and 10 μM SB431542 (Tocris) and was refreshed daily.

### Differentiation to mesendoderm and definitive endoderm

Endoderm differentiation was carried out using a protocol based on^45^, on laminin-521. Differentiation was started when the cells were 80–90% confluent with medium containing Roswell Park Memorial Institute (RPMI) 1640 supplemented with GlutaMAX (Thermo Fisher Scientific), 0.5% B27 supplement (Thermo Fisher Scientific), 100 ng/ml Recombinant Human/Mouse/Rat Activin A (R&D Systems) and 3 μM CHIR99021 (Stemgent). One day later the medium was changed to differentiation medium without CHIR99021 and the cells were cultured for two more days. For mesoderm induction, the same protocol was used but for only one day. To induce WNT and BMP4 activation we additionally supplemented the above protocol with CHIR99021 and/or Recombinant Human BMP4 Protein (R&D Systems) (Fig. 3A). To inhibit these pathways we used Recombinant Human Noggin Protein for BMP4 inhibition and/or XAV-939 for WNT inhibition (StemCell Technologies).

### Differentiation to hepatoblasts

Cells were plated in a ratio of 35,000 cells/cm^2^ a day prior to differentiation. The next day the medium was changed to Roswell Park Memorial Institute (RPMI) 1640 medium supplemented by GlutaMAX (Thermo Fisher Scientific), 100 ng/ml Recombinant Human/Mouse/Rat Activin A and 3 μM CHIR99021. One day later the medium was changed to differentiation medium without CHIR99021. From day 2 until day 8 the cells were cultured in Hepatoblast medium containing KnockOut D-MEM, 20% KnockOut Serum Replacement, 0.5X GlutaMAX, 1X 1% MEM Non-Essential Amino Acids (Thermo Fischer Scientific), 0.1 mM β-Mercaptoethanol (Sigma Aldrich), and 1% DMSO (Sigma Aldrich). The medium was refreshed every day.

### Differentiation to myogenic progenitors

Skeletal muscle differentiation was performed according to the protocol described in^46^, with few adjustments. Briefly, a total of 50,000 cells/cm^2^ were plated on a laminin-521 coated dishes. The next day, differentiation was induced by the use of 10 µM CHIR99021 in myogenic differentiation medium consisting of DMEM-F12, 1 × ITS-X, 100 U/ml Pen/Strep and 1 × Glutamine (all from Thermo Fischer Scientific) for 2 days. Subsequently, the CHIR99021 was replaced by 20 ng/ml FGF2 (Prepotech) for the following 10 days. Medium was refreshed daily.

### Differentiation to embryoid bodies

hESC cultured on laminin-521 (LN521, Biolamina) were passaged 2 days before embryoid body formation leading to a final confluence of 60–80%. Cells were harvested with TrypLE Express (Thermofisher Scientific) and resuspended in APEL medium (STEMCELL technologies) with 100U/ml Pen/Strep (Thermofisher Scientific) and 10 μM ROCKi (Sigma Aldrich). They were seeded in round-bottom non-treated 96-well plates with a final concentration was 5000 cells/well. The forming EBs were cultured for ten days in neutral conditions containing APEL medium, which was refreshed every second day. The protocols is described in detail in Dziedzicka et al.^47^.

### Quantitative real-time PCR

RNA extraction was performed with the QIAGEN mini or micro RNAeasy kit, cDNA conversion was performed with the GE-healthcare cDNA synthesis kit, following the manufacturers’ instructions. qRT-PCR was carried out on a ViiA 7 thermocycler (Thermo Fisher Scientific) and using standard protocol as provided by the manufacturer. The expression of the target genes was normalized to two house-keeping genes: UBC and GUSB, and all results are plotted as relative quantifications to either the expression in the genetically normal cells (VUB03_S1) or to untreated cells. Details on the probes, assays and primers are listed in the Supplementary Table S7.

### Immunostaining

Immunostaining for neuroectoderm, mesoderm, definitive endoderm and hepatoblasts was carried out on cells fixed and permeabilized with 4% paraformaldehyde (PFA) and 100% Methanol (Sigma-Aldrich), and blocked with 10% Fetal Bovine Serum (Thermo Fischer Scientific). For the myogenic progenitor differentiation cells were fixed with (PFA) for 10 min at room temperature and permeabilized using 0.3% Triton-X (Sigma-Aldrich) and blocked with 3% BSA, 0.1% Tween in PBS. The list with antibodies and manufacturers can be found in the Supplementary Table S8. Primary antibodies were incubated overnight at 4^0^C, secondary antibodies were incubated for 2–3 h at room temperature. Nuclear staining was performed with Hoechst 33342 (Thermo Fischer Scientific). Imaging was performed on a LSM800 confocal microscope (Carl Zeiss), and cell counts were done using the Zen 2 (blue edition) imaging software.

### RNA sequencing

150 ng of RNA was used to perform an Illumina sequencing library preparation using the QuantSeq 3′ mRNA-Seq Library Prep Kits (Lexogen) according to manufacturer's protocol. During library preparation 17 PCR cycles were used. Libraries were quantified by qPCR, according to Illumina's protocol 'Sequencing Library qPCR Quantification protocol guide', version February 2011. A High sensitivity DNA chip (Agilent Technologies) was used to control the library's size distribution and quality. Sequencing was performed on a high throughput Illumina NextSeq 500 flow cell generating 75 bp single reads. All data has been deposited on Gene Expression Omnibus repository with accession number GSE134454. The reads were mapped against the Genome Reference Consortium Human Build 38 patch release 10 (GRCh38.p10)^48^ using STAR (version 2.5.3)^49^. RNA-Seq by Expectation Maximization (RSEM)^50^ software (version 1.3.0) was used to produce the count table. Of the 63,967 Ensembl’s genes, only the somatic and X chromosomes’ coding genes were considered (19,802 genes in total).

### RNA-seq analysis

The RNA-seq analysis was performed using the R software (version 3.6.1)^35^ with the edgeR^51^ and DESeq2^52^ libraries. Only genes with a count per million (cpm) greater than 1 in at least two samples were considered. The raw counts were normalized using the trimmed mean of M values^53^ (TMM) algorithm. The normalized counts were then transformed in a log_2_ fold-change (log_2_FC) table with their associated statistics, p-value and false discovery rate (FDR). Genes with a |log_2_FC|> 1 and a FDR < 0.05 were considered as significantly differentially expressed. A |log_2_FC|> 1 means at least two times more or two times less transcript in the mutant group in comparison to the wild-type group.

The data was represented using a heatmap with an unsupervised hierarchical clustering and a principal component analysis using all expressed coding genes. The plots were generated using R^35^. Ingenuity Pathway Analysis^36^ (QIAGEN Inc.) was used for the pathway analysis based on the differential gene expression between groups. The pathway enrichment analysis was done using DAVID 6.8^54,55^. The top-1000 genes with the highest log_2_FC in absolute value were selected. In the different categories, only the GO terms were taken for account, only terms with p-value < 0.05 were considered relevant.

### Array comparative genomic hybridization (aCGH)

Oligonucleotide aCGH was carried out based on the protocol provided by the manufacturer (Agilent Technologies). A total of 400 ng of DNA was labeled with Cy3 while the reference DNA (Promega) was labelled with Cy5. The samples are hybridized on the microarray slide (4 × 44 K Human Genome CGH Microarray, Agilent Technologies). The slides were scanned using an Agilent dual laser DNA microarray scanner G2566AA. Only arrays with a SD. ≤ 0.20, signal intensity > 50, background noise < 5 and a derivative log-ratio < 0.2 were taken into account. Cutoff values were set at three consecutive probes with an average log_2_ ratio over 0.3 for gains and of -0.45 for loss.

### Exome sequencing

DNA was isolated following the manufacturers’ instructions (QIAGEN, Dneasy blood & tissue kit). DNA library preparation was performed with the Kapa HyperPrep kit (Roche, Basel, Switzerland), followed by exome target capturing with the SeqCap EZ exome probes V3 kit (Roche, Basel, Switzerland). Cluster generation was carried out on a cBOT system (Illumina, San Diego, CA) and samples were subsequently sequenced (paired end, 2 × 250 bp) on a HiSeq1500 machine (Illumina, San Diego, CA). Bio-informatic analysis of the data was done with an in-house developed pipeline based on Picard Tools (Broad Institute, Cambridge, MA), the BWA aligner^56^, GATK (Broad Institute, Cambridge, MA) and Alamut Batch (Interactive BioSoftware, Rouen, France). For the variant filtering, we used the software package Highlander (https://sites.uclouvain.be/highlander/) and used the BAM files as input.

## Supplementary Information

Supplementary Information
